# Different Relationships between Temporal Phylogenetic Turnover and Phylogenetic Similarity and in Two Forests Were Detected by a New Null Model

**DOI:** 10.1371/journal.pone.0095703

**Published:** 2014-04-18

**Authors:** Jian-Xiong Huang, Jian Zhang, Yong Shen, Ju-yu Lian, Hong-lin Cao, Wan-hui Ye, Lin-fang Wu, Yue Bin

**Affiliations:** 1 Key Laboratory of Vegetation Restoration and Management of Degraded Ecosystems, South China Botanical Garden, Chinese Academy of Sciences, Guangzhou, China; 2 University of Chinese Academy of Sciences, Beijing, China; 3 Department of Renewable Resources, University of Alberta, Edmonton, Alberta, Canada; Institute of Botany, Chinese Academy of Sciences, China

## Abstract

**Background:**

Ecologists have been monitoring community dynamics with the purpose of understanding the rates and causes of community change. However, there is a lack of monitoring of community dynamics from the perspective of phylogeny.

**Methods/Principle Findings:**

We attempted to understand temporal phylogenetic turnover in a 50 ha tropical forest (Barro Colorado Island, BCI) and a 20 ha subtropical forest (Dinghushan in southern China, DHS). To obtain temporal phylogenetic turnover under random conditions, two null models were used. The first shuffled names of species that are widely used in community phylogenetic analyses. The second simulated demographic processes with careful consideration on the variation in dispersal ability among species and the variations in mortality both among species and among size classes. With the two models, we tested the relationships between temporal phylogenetic turnover and phylogenetic similarity at different spatial scales in the two forests. Results were more consistent with previous findings using the second null model suggesting that the second null model is more appropriate for our purposes. With the second null model, a significantly positive relationship was detected between phylogenetic turnover and phylogenetic similarity in BCI at a 10 m×10 m scale, potentially indicating phylogenetic density dependence. This relationship in DHS was significantly negative at three of five spatial scales. This could indicate abiotic filtering processes for community assembly. Using variation partitioning, we found phylogenetic similarity contributed to variation in temporal phylogenetic turnover in the DHS plot but not in BCI plot.

**Conclusions/Significance:**

The mechanisms for community assembly in BCI and DHS vary from phylogenetic perspective. Only the second null model detected this difference indicating the importance of choosing a proper null model.

## Introduction

A central challenge in community ecology is to understand community dynamics [1], [2]. Community dynamics involve processes including birth, death, immigration and emigration that can be affected by various processes such as seed dispersal [2], habitat selection [3], [4], competition [5], disturbance [6], [7], or stochastic processes [2]. Ecologists have been documenting community dynamics, such as species-specific mortality rates, recruitment rates, population growth rates and the change of species composition, in many forest communities providing basic knowledge for biodiversity maintenance [8]–[10]. However, how species composition changes along a temporal axis (community dynamics) still remains poorly studied.

Community dynamics can results from both deterministic niche-based mechanisms [11] and stochastic mechanisms which consider only dispersal and ecological drift [2]. As species names do not convey critical information regarding the ecological and evolutionary similarity of species [12]–[15], exploring changes in taxonomic composition of species alone cannot distinguish deterministic mechanisms from stochastic processes [16]. A complete turnover in species composition of a community could result in a functionally analogous community, a directional change in the functional composition of a community, or changes in the community composition that are random with respect to species functions [14]. Thus, to make robust inferences, it is critical that the analysis of community turnover goes beyond traditional approaches of analyzing turnover in species composition by incorporating information pertaining to the ecological and evolutionary similarity of species [16].

With the increasing availability of phylogenetic data, computing power, and informatic tools, ecologists have witnessed a rapid expansion of studies that apply phylogenetic data and methods to community ecology (reviewed by Cavender-Bares et al. [17]). However, how phylogenetic similarity will influence phylogenetic temporal turnover is not well understood.

Two types of deterministic processes predict different phylogenetic temporal turnover when phylogenetic similarity is considered. Negative phylogenetic density dependence suggests that an individual surrounded with phylogenetically related neighbors will exhibit higher mortality than the reverse because they are likely susceptible to the same pathogens, fungi, etc [18], [19]. For the same reason, the empty space produced by the death of an individual will likely be occupied by a phylogenetically unrelated species in the species pool. In other words, if co-occurring species are closely related in phylogeny, negative phylogenetic density dependence predicts a high phylogenetic temporal turnover (positive relationship between phylogenetic temporal turnover and phylogenetic similarity). Alternatively, abiotic filtering, also known as species sorting, predicts species with functional traits incompatible with their environment experience elevated rates of mortality [20] which results in phylogenetic clustering [21] if functional traits exhibit a phylogenetic signal (Close phylogenetic related species have similar functional traits) [22]. Thus, given the dominance of habitat filtering, a community with phylogenetically unrelated species is expected to experience higher phylogenetic temporal turnover, i.e. a negative relationship between temporal phylogenetic turnover and phylogenetic similarity of co-occurring species.

To quantify temporal phylogenetic turnover, a few metrics have been developed [23]. However, the raw level of these metrics does not allow for making inferences regarding the cause for the observed phylogenetic turnover. Swenson et al. [16] proposed to randomize species name across a potential species pool as a null model for measuring the temporal phylogenetic turnover expected by chance. This null model has merits such as maintaining the observed species abundance, species richness, species occupancy rates, and species turnover through time. When applied to phylogenetic turnover through time, however, it implicitly assumes that all species have identical dispersal capability, immigrant history and survival rate across all life stages. These assumptions seem unrealistic to a natural plant community. For example, both dispersal ability and survival rate have a wide variation among coexisting species [24], [25]; additionally, species identity, survival rate also seems to be influenced by size [26], [27]. Appropriately considering these differences among species in the null model can improve inference for the cause of phylogenetic turnover through time.

In this study, we investigated phylogenetic turnover through time in two long-term forest dynamic plots using Swenson et al.'s null model [16] and a proposed new null model. In the new null model careful consideration of the difference in dispersal ability among species, the differences in survival rate both among species and life stages was given. How phylogenetic similarity of coexisting species affected phylogenetic turnover through time was tested. To better understand their relationship, the significance of phylogenetic signal for a set of important functional traits was tested.

## Materials and Methods

### Study sites

In this study, data was collected from two long-term forest plots: a 50-hectare tropical lowland moist forest plot in the Barro Colorado Island (BCI), Panama (9°10′N, 79°51′W) [28], and a 20-hectare subtropical forest plot in the Dinghushan (DHS) National Natural Reserve in southern China (23°19′N, 112°30′E). In each plot, all free standing woody stems with ≥1 cm diameter at breast height (DBH) or 1.3 m from the ground [29] were tagged, mapped, measured for DBH, identified to species level, and recorded for survival status during re-census.

To detect temporal changes in species composition, the most recent censuses years (2005 and 2010) were selected for both sites. In BCI, a total of 208,371 living individuals were recorded in 2005, including 297 species belonging to 182 genera and 59 families. In 2010, the number of individuals decreased to 207,200, with 30,414 deaths and 29,243recruits, including 7 lost species and 3 recruited species. Eight traits for the species in BCI were available, including maximum height, leaf area, leaf percentage carbon, leaf percentage nitrogen, leaf percentage phosphorus, seed mass and wood density. Phylogenetic signal for these eight functional traits had been tested in previous studies [16]. In DHS, the number of living individuals was 71,462 in 2005, including 195 species belonging to 114 genera and 54 families. In 2010, it decreased to 60,072 individuals, with 18,072 deaths and 6,601 recruits, including 20 lost species and 3 recruited species. Seven traits for 137 species in DHS were collected. These traits included leaf area, specific leaf area, leaf thickness, leaf dry mass content, leaf chlorophyll, petiole dry weight/fresh weight and wood density. These traits were chosen as they represent fundamental axes of ecological strategy in trees [16].

### Phylogenetic tree construction

Although molecular phylogenies are currently available for most of species in these two plots [30], [31], 16 (8.2%) and 27 (8.8%) rare species were not available for DHS and BCI plots, respectively. Rare species have low abundances and are sensitive to changes in habitat conditions and micro-successive stages, and thus are worth considering in our analysis. Therefore, phylogenies constructed by Phylomatic were used so that all species in these two censuses for both of plots were considered. A Phylomatic phylogeny was constructed using Phylomatic mega-tree by inputting all the species in the species list from the censuses into the plant phylogeny database [32], [33]. Phylomatic uses the Angiosperm Phylogeny Group Classification as the backbone for mega-trees. Branch lengths of phylogenetic trees were adjusted using the BLADJ algorithm [21] with known molecular and fossil dates [34]. These dates are rough estimates, but they make the quality of phylogenetic analyses more robust than nodal distances [12].

### Quantifying phylogenetic turnover

In this study Rao's quadratic Entropy (RaoD) was used to quantify temporal phylogenetic turnover through time [35]. RaoD is widely used in quantifying beta-diversity because it measures differences of species relative abundance and species differences [35]–[38]. When RaoD is applied to quantify temporal phylogenetic turnover, RaoD is the expected phylogenetic difference of a quadrat between two census times:
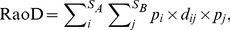
(1)where *S_A_* and *S_B_* are the numbers of species in a quadrat at time A and B, *p_i_* and *p_j_* are the relative abundances of species *i* and *j* in that quadrat, *d_ij_* is phylogenetic distance between species *i* and *j*.

Two null models were used to obtain the RaoD expected by chance. The first null model (NM-I) we used was to randomize species names across all species types in a phylogeny, as in Swenson et al. (2012) [16]. The second null model (NM-II) simulated the recruitment process when considering the variation in dispersal ability among species and simulated the mortality process when considering the variations in mortality both among species and among size classes within a species. To simulate the recruitment process, species were first classified into either tree or shrub. According to advice from experts in DHS plot, trees and shrubs with DBH≥15 and 5 cm in the first census were considered mature and potential parents for the recruits, respectively (Zhongliang Huang, personal communication). The nearest conspecific mature individual within 50 m of this recruit was considered its parent. The distance between a recruit and its parent was recorded as *Dp*. A potential parent was then randomly selected as a recruit from all of conspecific mature individuals in the plot. The coordinates for the location of this recruit was randomly generated from a circle, for which the position of the selected parent is its center and *Dp* is its radius, If a recruit has no possible parents within 50 m, its position was randomly generated within the bounds of the plot using uniform distribution [39]. To simulate mortality, we first grouped all individuals for each species in the first census into size classes by DBH of 1–2, 2–3, … 19–20, 20–25, 25–35, 35–45, 45–55 and ≥55 cm and calculated the survival rates for each of size class of each species. We then randomly assigned mortality to individuals in each size class for each species based on these species-specific and size-specific survival probabilities.

Randomized data sets were obtained for the second census of the BCI and the DHS plots using these two null models. For each plot, 499 such data sets were generated and calculated a RaoD for each of them. Then, the observed RaoD (RaoD.obz) for a quadrat was standardized to a standardized effect size (ses.RaoD) by the 499 null RaoDs (RaoD.null)as:
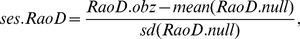
(2)where mean(RaoD.null) and sd(RaoD.null) are the mean and standard deviation of RaoD.null generated by the 499 simulated distributions. Thus, ses.RaoD can explain to what extent the observed temporal turnover is different from what expected by chance.

### Test of phylogenetic signal

The phylogenetic signal was examined among those species across the phylogeny using the statistic *K* which is the tendency of related species to resemble one another or the amount of phylogenetic signal [22]. *K* is close to zero when there is no phylogenetic signal for a functional trait. *K* is equal to one under a Brownian motion model, when traits change by small random amounts and at a constant rate through time. *K* is greater than one when close relatives are more similar than expected under Brownian motion evolution, indicating a strong phylogenetic signal for a functional trait. To evaluate the significance of the phylogenetic signal, a null expectation of *K* under no phylogenetic signal was generated by randomly shuffling the tips of the phylogeny 1000 times, and a probability that the observed *K* is higher than randomization is calculated. The probability generated indicates statistical significance of phylogenetic signal of a functional trait across a phylogeny.

### Statistical analysis

Each plot was divided into non-overlapping square quadrats with side lengths of 10, 20, 30, 40 and 50 m. The index ses.RaoD was used to quantify the temporal phylogenetic turnover against that expected by chance. Net phylogenetic relatedness index (NRI) was used to represent phylogenetic similarity of species in a quadrat [21]. NRI is the negative standardize effect size of mean phylogenetic distance, calculated as
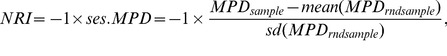
(3)where MPD_sample_ is observed MPD (mean of all pairwise phylogenetic distances between co-occurring taxa) for the quadrate, and mean (MPD_rndsample_) and sd (MPD_rndsample_) are mean and standard deviation of MPDs from 499 simulations. In each simulation, an independent swap algorithm randomization process was performed to generate the random assemblages [40]. This randomization process maintains species occurrence frequency and sample species richness as well.

A simultaneous autoregressive error model (SAR_err_) with 1 grid-cell lag-distance was used to fit the relationship between ses.RaoD and *NRI*. Spatial autocorrelation is a frequent phenomenon in ecological data and can affect estimates of model coefficients and inference from statistical models and SAR_err_ model is recommended when dealing with spatially autocorrelated species distribution data [41].

Spatial variations of ses.RaoD among quadrats at each of the five spatial scales explained by NRI and spatial structure represented by principal coordinates of neighbor matrices (PCNM) eigenfunctions were analyzed by variation partitioning [42], [43]. Partitioning was based on adjusted R^2^ statistic (*R^2^_a_*) in multiple linear regressions [44]. PCNM eigenfunctions describe all spatial scales that can be accommodated in the five quadrat sizes. They were obtained by principal coordinate analysis (PCoA) of a truncated geographic distance matrix among the sampling subplots. In the present study, all distances larger than those between the centers of diagonally adjacent subplots were replaced by four times their values before PCoA. A forward selection with permutation tests at 5% significance level based on R^2^ increases at each step was used to remove those PCNM eigenfunctions that were not significantly correlated with the detrended ses.RaoD. Before variation partitioning, ses.RaoD was detrended using third-degree orthogonal polynomials, and the residuals were retained for variation partitioning [45].

All statistical analyses were performed in the software R 2.15.2 [46]. NRI was calculated using “picante” package [47]. The SAR analyses were computed using “spdep” package [48], PNCM eigenfunctions and variation partitioning were computed using the “vegan” package [49], and forward selection was computed using “packfor” package.

## Results

In DHS, five of seven traits exhibit significant phylogenetic signals (*P*<0.05) (Table 1). In BCI, *K* values were all less than 0.1 (close to zero) for all of the tested eight functional traits [16], indicating that phylogenetic signal was little to null for the eight functional traits for tree species in the BCI plot. The results suggest that using phylogenetic distance as a surrogate for difference of functional traits is proper for species in DHS plot but not reliable for species in BCI plot.

**Table 1:** Test of phylogenetic signal in seven functional traits for species in the DHS plot.

Functional Trait	*K*	p-value
Leaf Area	0.134	0.956
Specific Leaf Area	0.481	0.002
Leaf thickness	0.402	0.06
Leaf dry mass content	0.488	0.001
Leaf chlorophyll	0.434	0.004
Petiole dry weight/fresh weight	0.448	0.013
Wood density	0.569	0.001

The *K* statistic proposed by Blomberg *et al.* (2003) indicates the amount of phylogenetic signal, and p-value indicates statistical significance of phylogenetic signal of a functional trait across a phylogeny, to which the observed phylogenetic signal is different from null model.

For both null models, spatial autocorrelation of residuals of models at five scales in the two plots were tested using Moran's I based on 999 random permutations [50]. Except at 50 m scale in DHS using NM-II, significant spatial autocorrelation was detected when simple linear regression was used but disappeared when SAR_err_ was used (see Table S1) indicating that ses.RaoD was significantly spatially autocorrelated and that the spatial autocorrelation in ses.RaoD was successfully removed using SAR_err_.

**Figure 1:**
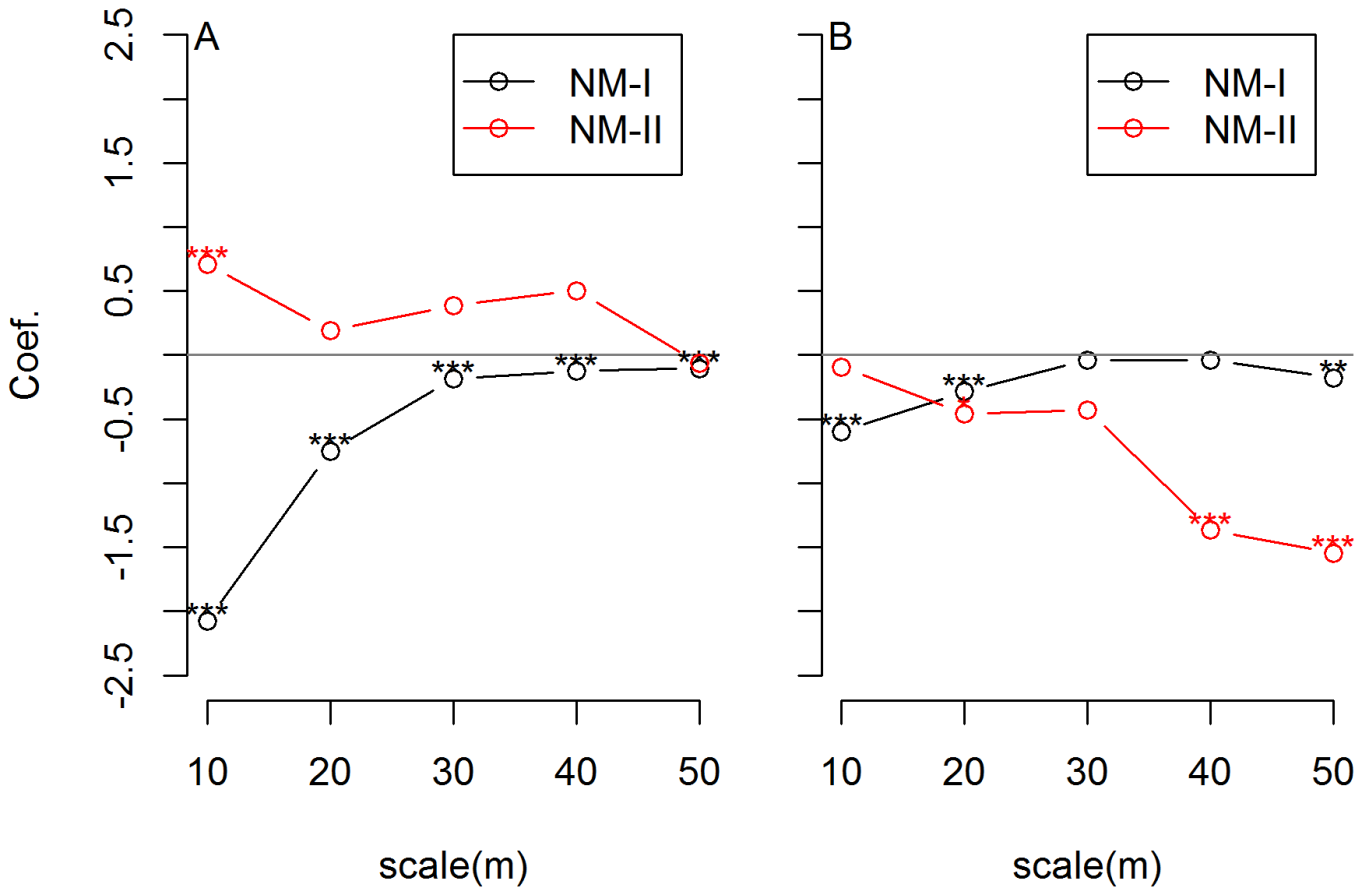
The relationship between phylogenetic similarity and phylogenetic turnover along five spatial scales for BCI and DHS. The relationship between phylogenetic similarity and phylogenetic turnover based on spatial simultaneous autoregressive error model across scales for the BCI (A) and DHS (B) plots. Y-axis is regression coefficient of ses.RaoD against NRI. The independent variable is phylogenetic diversity index NRI and the dependent variable is phylogenetic turnover ses.RaoD, which is the standardized effect size of RaoD of a null model. Null Model-I (NM-I) shuffles species names across all species in each plot, while Null Model-II (NM-II) randomizes the location of mortality according to species- and size-specific survival probability and the location of recruitment according to species-specific dispersal ability. The black line was from NM-I, and the red line from NM-II. ‘**’ indicates p value<0.001 and >0.0001. ‘***’ indicates p value<0.0001.

When NM-I was used to calculate ses.RaoD, results of SAR_err_ show that NRI was negatively correlated with phylogenetic turnover (evaluated by ses.RaoD) at all of five spatial scales in both of plots, suggesting that phylogenetically closely related species formed more temporally stable communities (Figure 1). When NM-II was used, there were markedly different results for the BCI and the DHS plots. In the BCI plot, the correlation between NRI and ses.RaoD was positive except at the largest scale but this relationship was significant only at the finest scale (Figure 1A). In contrast to the BCI plot, in DHS plot, NRI was negatively correlated with ses.RaoD at all scales, significant at the scales of 20, 40 and 50 m but not at scales of 10 m and 30 m (Figure 1B). The absolute values of regression coefficients increased with increasing scales except for 30 m. This trend was not observed with NM-I.

**Figure 2:**
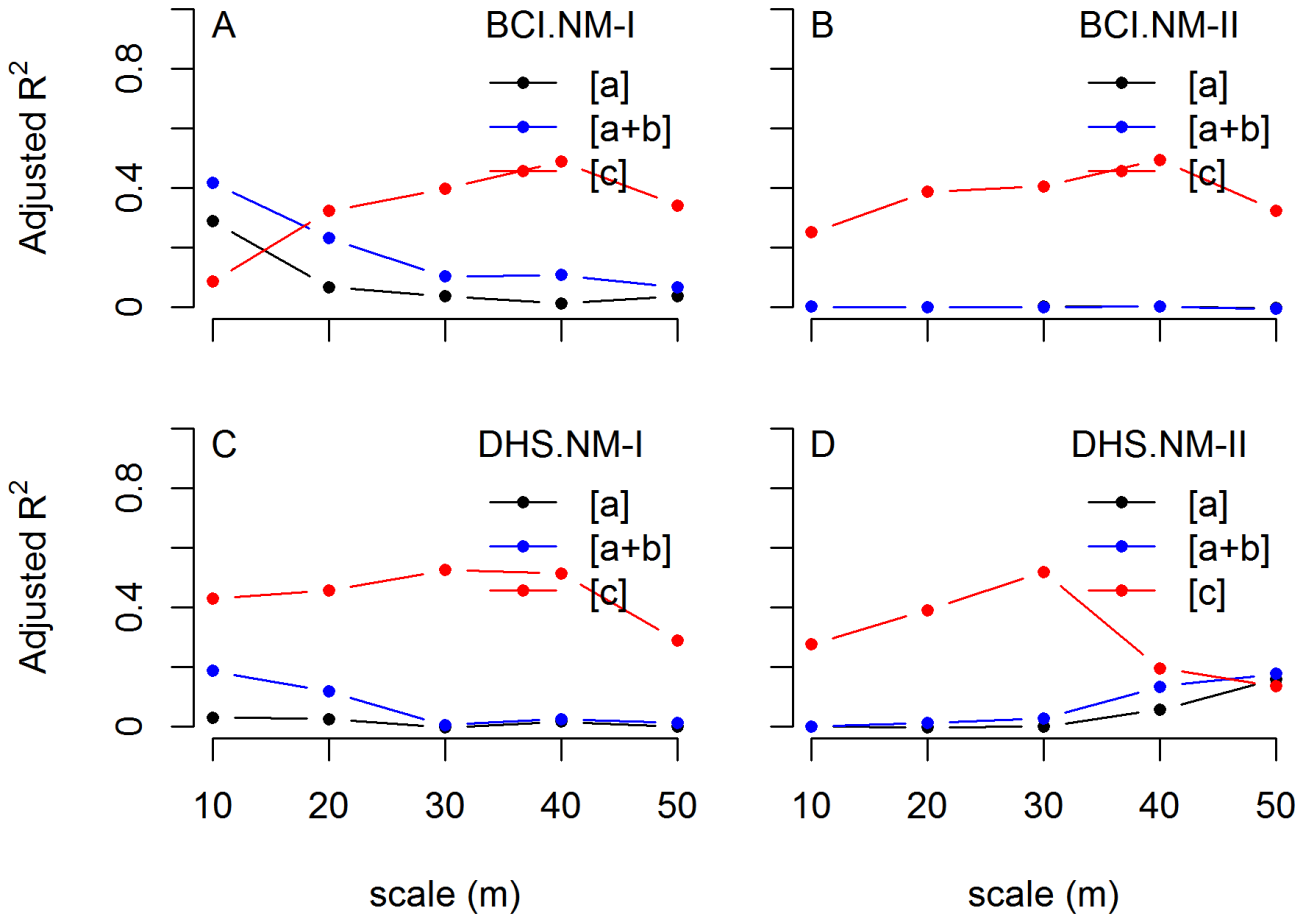
Results of variation partitioning along five spatial scales for BCI and DHS. The variation partitioning results for ses.RaoD using two Null models (NM-I and NM-II) for DHS and BCI plots along five spatial scales. [a] : the non-spatially structured part of variation explained by NRI, [a+b] : the total variation explained by NRI, [c] : the spatially structured variation not explained by NRI (the variance uniquely explained by PCNM eigenvectors). A) BCI plot using NM-I, B) BCI plot using NM-II, C) DHS plot using the NM-I, and D) DHS plot using the NM-II. Note that [a] and [a+b] are almost equal and are indistinguishable in panel A.

With NM-I, in the BCI plot, the proportion of variation of ses.RaoD explained by NRI totally in terms of [a+b] decreases from 41.7% at the 10 m scale to 6.8% at the 50 m scale (Figure 2A). In the DHS plot, the proportion of variance explained by NRI ranged from 0.6% to 18.7% (Figure 2C). No obvious trend was observed for the variation explained by NRI as scale increased, in contrast to the BCI plot.

NM-II showed a minimal contribution of NRI ([a+b]) to the variance of ses.RaoD in the BCI plot. PCNMs account for 25.1% to 49.6% of the variance at the five scales indicated by [c] (Figure 2B). In the DHS plot, the variation of ses.RaoD explained by NRI in terms of [a+b] increases from approximately 0% at 10 m scale to 17.7% at the 50 m scale (Figure 2D). In addition, for both plots, the proportion of variance explained by PCNMs increases at first and then decreases as scale increases. More details can be found in Table S2.

**Figure 3:**
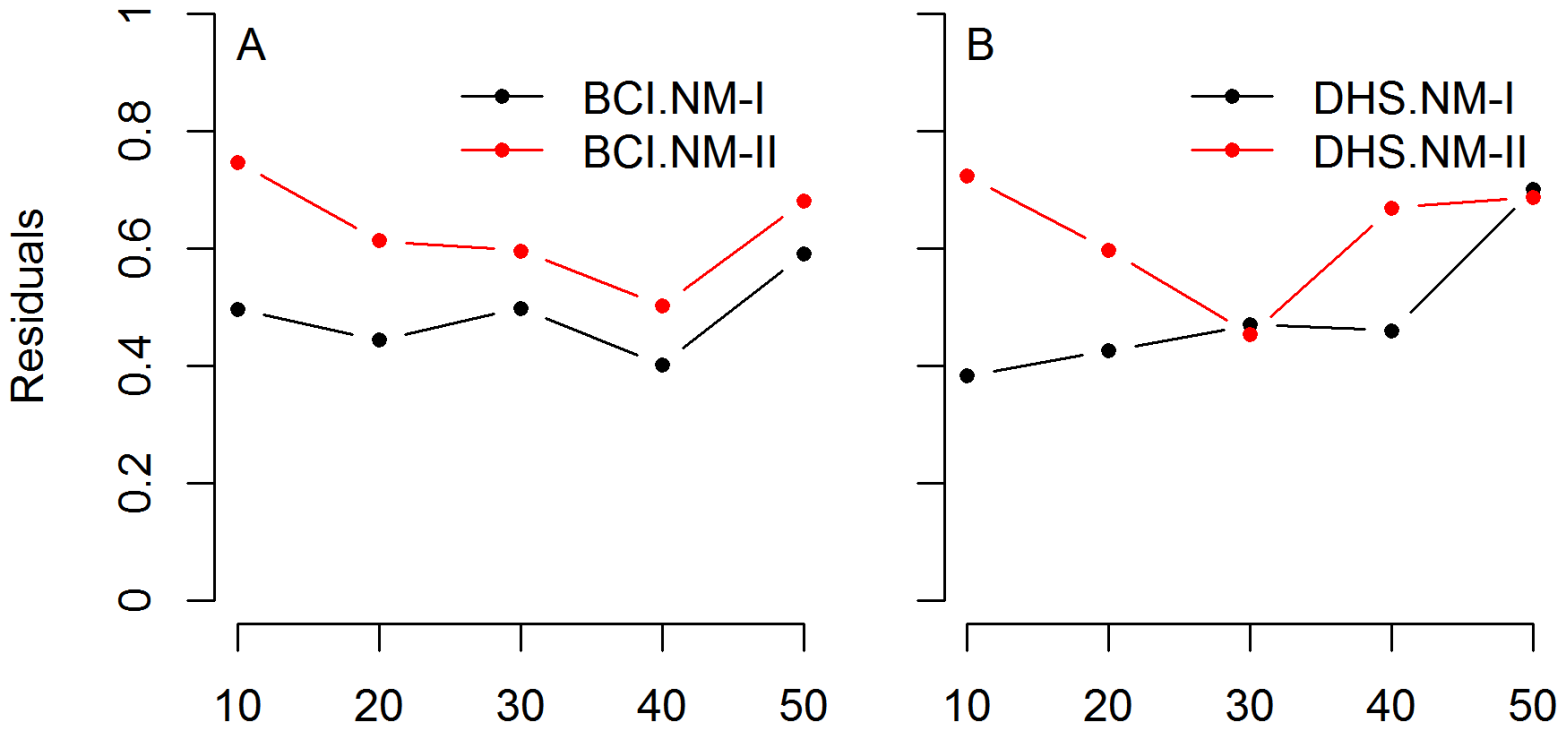
Comparing of residuals in variation partitioning between two null models. Comparing of residuals in variation partitioning between two Null models (NM-I and NM-II) along five spatial scales. A) BCI plot, B) DHS plot.

Comparison of residuals using variation partitioning analysis showed that the proportion of unexplained variations using NM-I were higher than using NM-II for eight of ten cases in the two plots, except for 30 and 50 m scales in DHS plot where the proportion of unexplained variations using NM-I was slightly lower than using NM-II (Figure 3).

## Discussion

To obtain the expected phylogenetic turnover by chance, a widely used method is to shuffle name of species across all tips of a phylogeny (NM-I) [12], [16], [51]. With NM-I, negative correlation between ses.RaoD and NRI were found in both BCI and DHS plots, suggesting that processes affecting phylogenetic turnover were generally similar in these two plots. This force could be abiotic filtering if species in the two plots represent niche conservatism. The importance of abiotic filtering is supported by previous studies in DHS where habitat conditions affect the distribution of 83% of species [52] and habitat factors explained 23.7% at the 10 m scale and 54.1% at the 100 m scale of the variation in species distribution [53]. However, in BCI, the main force for community structure may differ according to previous studies. Harms et al. [4] found that in the BCI plot, among 181 species, only 28 species were significantly associated with the most extensive habitat in the BCI plot, indicating a limited role of habitat condition for community structure. Hubbell [2] found that relative species abundance in the BCI plot could be fitted by neutral processes extremely well. In a comparative study, habitat factors were less important to species distribution in BCI than in DHS [54]. Taken together, the traditional null model generated results consistent with previous analyses in DHS plot but not with previous findings for the BCI plot. We speculated that the assumption of equal dispersal ability and survival rate of this model has reduced the ability to detect deterministic effect and led to this discrepancy.

To improve efficiency, the positions of the recruits were randomized based on distance to the nearest conspecific adult so that differences in dispersal ability and seedling survival rates can be considered to some degree. The occurrence of mortality was also randomized based on species-specific and size-specific survival rates in NM-II (the observed mortality of species at all size classes in BCI and DHS plots between 2005 and 2010 can be found in table S3 and table S4, respectively). Using NM-II, the direction of the correlation between NRI and ses.RaoD did not changed in DHS. In BCI, this correlation was significantly positive at the finest scale, positive but not significant at the three intermediate scales, and negative but not significant at the largest scale. A previous study found that phylogenetic similarity decreases with size at local spatial scale (10–15 m) in BCI [13], suggesting a larger phylogenetic turnover for phylogenetically close communities. This is consistent with the positive correlation between NRI and ses.RaoD found using NM-II. Therefore, using NM-II, the results for both the BCI and DHS plots were in agreement with previous studies.

As spatial scale increases, according to the well-established species-area relationship [55], species composition of a sampled community becomes more complete and community dynamics are likely to become more self-regulated [56], [57]. Thus, the proportion of variance explained by NRI was expected to increase with spatial scale. This expectation was supported using NM-II but not the traditional null model. Additionally, the unexplained fraction of phylogenetic distance using NM-I was higher than NM-II for most of cases. Therefore, we believe that NM-II is more appropriate than the null model traditionally used in related studies.

According to the analysis based on NM-II, phylogenetic similarity positively influences temporal turnover only at the finest scale in BCI plot. In other words, a quadrat in the BCI plot with distantly related species is more phylogenetically stable than with phylogenetically closely related species between the census periods of 2005 to 2010. As mentioned above, this positive correlation is supported by previous study in BCI [13]. This can be explained by phylogenetic density dependent effect. When this effect dominates, individuals are more likely to be eliminated from a quadrat (or to introduce a turnover) when they are surrounded by closely related neighbors. However, the partition results showed that the proportion of variance explained by NRI was almost zero for all of five scales in BCI plot, suggesting that the significant positive correlation between ses.RaoD and NRI at 10 m scale detected using SAR_err_ is weak.

In the DHS plot, there was a significant negative relationship between phylogenetic similarity and phylogenetic turnover at three of the five scales examined. This suggests that phylogenetically related communities in the DHS plot are more stable than phylogenetically unrelated communities, in contrast to the BCI plot. Considering that there is significant phylogenetic signal for most of tested functional traits, abiotic filtering is a reasonable explanation for this pattern. For given a habitat condition, species with certain traits that survive under that habitat condition should be selected by the habitat while other species that are not suitable are excluded. Therefore, different from phylogenetic density dependent effect, abiotic filtering accumulates species that are functionally similar, or phylogenetically close if functional traits show phylogenetic signal. Therefore, abiotic filtering expects communities with low NRI to have large phylogenetic turnover because a large proportion of the species in these communities are to be excluded by the habitat condition, leaving space for recruits that are favored by the habitat condition. Thus, the negative correlation between NRI and ses.RaoD indicates the importance of abiotic filtering, consistent with previous studies conducted in the same plot [52], [53]. In the DHS plot, the proportion of variance in ses.RaoD explained by NRI increases from about 0% to 17.1% as the scale increased, which suggests that abiotic filtering have played an increasingly important role in the plot as scale increased.

In both plots, the proportion of variance of ses.RaoD explained by PCNMs is much higher than by NRI, suggesting the importance of spatial autocorrelation for phylogenetic turnover. In this study, spatial autocorrelation of temporal turnover consists of spatial autocorrelation of the occurrence of recruitment and mortality. On the one hand, because of dispersal limitation, neighbor quadrats are more likely to share similar species composition in the seed pool than quadrats that are spatially separated [58], which leads to spatial autocorrelation for the phylogenetic structure of recruits. On the other hand, limited dispersal and patchy habitat condition together results in autocorrelated species distribution [4], [59], [60]. This autocorrelation in species distribution can turn into autocorrelated phylogenetic structure of dead individuals because mortality rates also differ among species (see [61]). The strong autocorrelation in phylogenetic turnover indicates the importance of seed dispersal and survival probability and possibly other small-scale processes.

Although results using NM-II were more reasonable than using NM-I in this study, there were still limitations in the analysis. For example, when simulating the recruitment process, diameters of 15 cm and 5 cm were arbitrarily used to define mature individuals for the tree and shrub species respectively. About 60% recruits (4,683 of 6,679 in DHS plot and 19,472 of 36,525 in BCI plot) did not have a possible parent plant within 50 m. When simulating the mortality process, we took into account only the variation in mortality among size classes and among species. Other factors like habitat conditions that influence mortality risk were not considered. We believe the result can be improved by a more realistic procedure in future studies.

In conclusion, we found large spatial autocorrelation in phylogenetic turnover in both plots, indicating the importance of spatially structured processes in both of these two plots. Phylogenetic relatedness positively and negatively associated with phylogenetic turnover in BCI and DHS, respectively, implying different conservative strategies for species at these two sites.

## Supporting Information

Table S1 Spatial autocorrelation of residuals of models at five scales in the two plots were tested using Moran's I based on 999 random permutations. P value less than 0.01 indicates significant spatial autocorrelation is detected using Moran's I.
(DOC)

Table S2 Results of variation partitioning for ses.RaoD by NRI and PCNMs. NM-I, null model I, that considers deaths, recruits and the unvaried individuals separately. NM-II, null model II, that shuffles species names across all species in each of the plots.
(DOC)

Table S3 Mortality of species for DBH size classes in BCI plot.
(XLS)

Table S4 Mortality of species for DBH size classes in DHS plot.
(XLS)
