# Supplementary material for: Different Relationships between Temporal Phylogenetic Turnover and Phylogenetic Similarity and in Two Forests Were Detected by a New Null Model
Source: PLoS One. 2014 Apr 18;9(4):e95703. doi: 10.1371/journal.pone.0095703 (PMC3991709; doi:10.1371/journal.pone.0095703)
Supplement: Table S1 — Spatial autocorrelation of residuals of models at five scales in the two plots were tested using Moran's I based on 999 random permutations. P value less than 0.01 indicates significant spatial autocorrelation is detected using Moran's I. (DOC) [file pone.0095703.s001.doc]

**Table S1.** spatial autocorrelation of residuals of models at five scales in the two plots were tested using Moran’s I based on 999 random permutations. P value less than 0.01 indicates significant spatial autocorrelation is detected using Moran’s I.

| Plot | Null Model | scale (m) | linear regression | | SAR_err_ | |
| --- | --- | --- | --- | --- | --- | --- |
|  |  |  | Moran's I | P Value | Moran's I | P Value |
| BCI | NM-I | 10 | 0.2377 | 0.001 | -0.032 | 0.994 |
|  |  | 20 | 0.4227 | 0.001 | -0.0766 | 0.999 |
|  |  | 30 | 0.4265 | 0.001 | -0.0802 | 0.995 |
|  |  | 40 | 0.4678 | 0.001 | -0.0491 | 0.864 |
|  |  | 50 | 0.5688 | 0.001 | -0.1126 | 0.973 |
|  | NM-II | 10 | 0.297 | 0.001 | -0.0334 | 0.999 |
|  |  | 20 | 0.2945 | 0.001 | -0.0143 | 0.764 |
|  |  | 30 | 0.3095 | 0.001 | -0.0313 | 0.823 |
|  |  | 40 | 0.3409 | 0.001 | -0.0206 | 0.642 |
|  |  | 50 | 0.309 | 0.001 | -0.0342 | 0.713 |
| DHS | NM-I | 10 | 0.4342 | 0.001 | -0.1081 | 0.999 |
|  |  | 20 | 0.5112 | 0.001 | -0.1163 | 0.998 |
|  |  | 30 | 0.6328 | 0.001 | -0.0811 | 0.935 |
|  |  | 40 | 0.5667 | 0.001 | -0.0926 | 0.885 |
|  |  | 50 | 0.6144 | 0.001 | -0.0754 | 0.789 |
|  | NM-II | 10 | 0.117 | 0.001 | -0.0073 | 0.666 |
|  |  | 20 | 0.1922 | 0.001 | -0.0107 | 0.603 |
|  |  | 30 | 0.2164 | 0.001 | -0.0038 | 0.487 |
|  |  | 40 | 0.1488 | 0.008 | -0.0037 | 0.486 |
|  |  | 50 | 0.002 | 0.426 | -0.0004 | 0.464 |
