# Supplementary material for: Different Relationships between Temporal Phylogenetic Turnover and Phylogenetic Similarity and in Two Forests Were Detected by a New Null Model
Source: PLoS One. 2014 Apr 18;9(4):e95703. doi: 10.1371/journal.pone.0095703 (PMC3991709; doi:10.1371/journal.pone.0095703)
Supplement: Table S2 — Results of variation partitioning for ses.RaoD by NRI and PCNMs. NM-I, null model I, that considers deaths, recruits and the unvaried individuals separately. NM-II, null model II, that shuffles species names across all species in each of the plots. (DOC) [file pone.0095703.s002.doc]

**Table S2**. Results of variation partitioning for ses.RaoD by NRI and PCNMs. NM-I, null model I, that considers deaths, recruits and the unvaried individuals separately. NM-II, null model II, that shuffles species names across all species in each of the plots.

| Plot | null  model | Cell size  (m) | adjusted R2 statistics, R2a | | | | | | | $\frac{[b]}{[a+b]}\%$ |
| --- | --- | --- | --- | --- | --- | --- | --- | --- | --- | --- |
|  |  |  | n | [a] | [b] | [c] | [d] | [a+b] | [b+c] |  |
| BCI | NM-I | 10 | 5000 | 0.002 | 0 | 0.251 | 0.747 | 0.002 | 0.251 | 0 |
|  |  | 20 | 1250 | 0 | 0 | 0.387 | 0.613 | 0 | 0.387 | - |
|  |  | 30 | 528 | 0.002 | -0.002 | 0.405 | 0.596 | 0 | 0.403 | - |
|  |  | 40 | 300 | 0.002 | 0.001 | 0.495 | 0.502 | 0.003 | 0.496 | 33.3 |
|  |  | 50 | 200 | -0.003 | -0.002 | 0.324 | 0.681 | -0.005 | 0.322 | - |
|  | NM-II | 10 | 5000 | 0.288 | 0.13 | 0.086 | 0.496 | 0.417 | 0.216 | 31.1 |
|  |  | 20 | 1250 | 0.068 | 0.164 | 0.323 | 0.444 | 0.232 | 0.487 | 70.6 |
|  |  | 30 | 528 | 0.038 | 0.066 | 0.397 | 0.498 | 0.104 | 0.463 | 63.1 |
|  |  | 40 | 300 | 0.014 | 0.094 | 0.489 | 0.402 | 0.109 | 0.584 | 86.9 |
|  |  | 50 | 200 | 0.037 | 0.031 | 0.342 | 0.59 | 0.068 | 0.373 | 45.3 |
| DHS | NM-I | 10 | 2000 | 0 | 0 | 0.276 | 0.724 | 0 | 0.276 | - |
|  |  | 20 | 500 | -0.001 | 0.014 | 0.391 | 0.597 | 0.013 | 0.405 | 100 |
|  |  | 30 | 208 | 0.001 | 0.026 | 0.519 | 0.454 | 0.027 | 0.545 | 96.3 |
|  |  | 40 | 120 | 0.057 | 0.078 | 0.196 | 0.669 | 0.135 | 0.274 | 57.8 |
|  |  | 50 | 80 | 0.159 | 0.018 | 0.136 | 0.687 | 0.177 | 0.154 | 10.2 |
|  | NM-II | 10 | 2000 | 0.031 | 0.156 | 0.43 | 0.383 | 0.187 | 0.586 | 83.4 |
|  |  | 20 | 500 | 0.026 | 0.092 | 0.456 | 0.426 | 0.118 | 0.548 | 77.9 |
|  |  | 30 | 208 | -0.002 | 0.008 | 0.525 | 0.47 | 0.006 | 0.533 | 100 |
|  |  | 40 | 120 | 0.017 | 0.009 | 0.514 | 0.46 | 0.026 | 0.523 | 36.4 |
|  |  | 50 | 80 | 0.001 | 0.011 | 0.288 | 0.7 | 0.012 | 0.299 | 90.8 |

Notes: [a]–[d] (adjusted R^2^ statistics, R^2^_a_): [a] = variation explained by the NRI and not spatially structured, [b] = variation explained by NRI and spatially structured, [c] = spatially structured variation not explained by NRIs, [d] = residuals. Fraction [b] is the intersection of variation explained by linear models of NRI and spatial structured factors. Principal coordinates of neighbor matrices (PCNM) eigenfunctions were the explanatory variables used to compute fractions. Proportion of variation explained by NRI that are spatially structured is indicated by ([b]/[a+b])%. If [b] or [a+b] is less than zero, zero is used in calculating the value.
